# Stochastic gradient boosting frequency-severity model of insurance claims

**DOI:** 10.1371/journal.pone.0238000

**Published:** 2020-08-31

**Authors:** Xiaoshan Su, Manying Bai

**Affiliations:** Department of Finance, Beihang University, Beijing, China; Tongii University, CHINA

## Abstract

The standard GLM and GAM frequency-severity models assume independence between the claim frequency and severity. To overcome restrictions of linear or additive forms and to relax the independence assumption, we develop a data-driven dependent frequency-severity model, where we combine a stochastic gradient boosting algorithm and a profile likelihood approach to estimate parameters for both of the claim frequency and average claim severity distributions, and where we introduce the dependence between the claim frequency and severity by treating the claim frequency as a predictor in the regression model for the average claim severity. The model can flexibly capture the nonlinear relation between the claim frequency (severity) and predictors and complex interactions among predictors and can fully capture the nonlinear dependence between the claim frequency and severity. A simulation study shows excellent prediction performance of our model. Then, we demonstrate the application of our model with a French auto insurance claim data. The results show that our model is superior to other state-of-the-art models.

## Introduction

Insurance claims modeling is a topic of great concern in non-life insurance. The model helps an insurer accurately estimate potential loss and make appropriate actuarial decisions. Specifically, the model enables an insurer to set a fair premium for each individual policy. It is important to charge the policyholder with a fair premium. For instance, Dionne, Gouriéroux, and Vanasse [1] point out that in auto insurance, if an insurer charges too little for young drivers and too much for old drivers, young drivers will be attracted while old drivers will switch to competitors. Then, the insurer loses profitable and gain underpriced policies, both resulting in economic losses. Further, the model helps the insurer determine a suitable level of risk capital. The underestimation of loss can make the insurer not hold enough risk capital and hence raise bankruptcy risk. In contrary, the overestimation can reduce liquid capital of the insurer and then hamper business expansion. Thus, an accurate model of insurance claims is significant to competency and profits of an insurer.

The frequency-severity model is a standard model of insurance claims, which separately models the claim frequency and average claim severity. The claim frequency examines the number of claims and the average claim severity takes account of the average amount of claims conditional on occurence. The claim frequency and severity depend on characteristics of an individual policy. For instance, in auto insurance, the characteristics include the age, gender and motor vehicle record points of the policyholder, per capital income and population density of the policyholder’s residential area, age and model of the vehicle, etc. Thus, there is a need of predictive models. The traditional frequency-severity model uses generalized linear models (GLM) for modeling the claim frequency and severity. The frequency part often uses Poisson or negative binomial regressions and the severity part uses gamma or inverse Gaussian regressions. There is a large literature extending the model to capture different features of the data. For instance, multivariate models can give a joint analysis of the frequency or severity at different levels of classification. Anastasopoulos, Shankar, Haddock, and Mannering [2] introduce a multivariate Tobit model to study accident rates categorized by severities. The conditional autoregressive model can be used for accommodating spatial correlation. Huang, Song, Xu, Zeng, Lee, and Abdel-Aty [3] develop a macro-level Bayesian spatial model with conditional autoregressive prior and a micro-level Bayesian spatial joint model for predicting the claim frequency. Zeng, Wen, Wong, Huang, Guo, and Pei [4] use a bivariate conditional autoregressive model to simultaneously analyze daytime and nightime claim frequencies. Aguero-Valverde [5] introduces a multivariate conditional autoregressive model to estimate excess claim frequencies at different severity levels. Generalized linear mixed models and the other random parameters models can be used to capture unobserved heterogeneity across observations. Barua, El-Basyouny, and Islam [6] develop a multivariate random parameters conditional autoregressive model to predict claim frequencies. Zeng, Wen, Huang, Pei, and Wong [7] propose a multivariate random parameters Tobit model to analyze accident rates by severity. Zeng, Guo, Wong, Wen, Huang, and Pei [8] introduce a multivariate random parameters spatio-temporal Tobit model to accommodate spatio-temporal correlation and interaction. Dong, Ma, Chen, and Chen [9] use a mixed logit model to examine the difference of single-vehicle and multivehicle accident probabilities. Chen, Chen, and Ma [10] adopt a mixed logit model to analyze the hourly accident probability for highway segments. Chen, Song, and Ma [11] develop a random parameters bivariate ordered probit model to investigate the injury severity of the two drivers involved in the same crash.

However, there are two major limitations in the frequency-severity model. First, the model has a linear predictor form. In practice, there are nonlinear effects from predictors. For instance, in auto insurance, the nonlinear relation between the claim severity and the insured’s age is well documented (Frees, Shi, and Valdez [12]). Generalized additive models (GAM) developed in Hastie and Tibshirani [13] and popularized by Wood [14] overcome the restrictive linear form by modeling continuous variables with smooth functions estimated from data. However, the additive form of GAM models can’t automatically capture complex interactions among predictors. Though interaction terms can be manually added to the structure of the model, identifying interactions terms can be tedious when many predictors are involved. Missing important interactions can reduce prediction accuracy. Second, the standard frequency-severity model assumes an independent relation between the claim frequency and severity. However, in practice, the claim frequency and severity are often dependent. For instance, in auto insurance, the claim frequency and severity are often negatively correlated (Gschlößl and Czado [15]). Home insurance claims due to natural hazard such as earthquake or flood are both large and frequent in affected areas. Frees, Gao, and Rosenberg [16] also point out that the claim frequency has a significant effect on the claim severity for outpatient expenditures. Gschlößl and Czado [15], Frees, Gao, and Rosenberg [16], Erhardt and Czado [17], Shi, Feng, and Ivantsova [18] and Garrido, Genest, and Schulz [19] capture the dependence between the claim frequency and severity by treating the claim frequency as a predictor variable in the regression model for the average claim severity. Shi, Feng, and Ivantsova [18] show that the predictor method applied to the GLM frequency-severity model can only measure a linear relation between the claim frequency and severity. Czado, Kastenmeier, Brechmann, and Min [20], Krämer, Brechmann, Silvestrini, and Czado [21] and Shi, Feng, and Ivantsova [18] model the joint distribution of the claim frequency and average claim severity through copulas. However, popular copulas, such as elliptical and Archimedean copulas, can only capture the symmetric or limited dependence structures. The multivariate conditional autoregressive model (Aguero-Valverde [5]) with its random parameters version (Barua, El-Basyouny, and Islam [6]) and the multivariate Tobit model (Anastasopoulos, Shankar, Haddock, and Mannering [2]) with its random parameters version (Zeng, Wen, Huang, Pei, and Wong [7]) and its random parameters spatio-temporal version (Zeng, Guo, Wong, Wen, Huang, and Pei [8]) accommodate the correlation between the claim frequency and severity by modeling claim frequencies or accident rates at different severity levels. But the usage of finitely many severity levels only partially captures the dependence between the claim frequency and severity. Thus, there is a need to develop a data-driven dependent frequency-severity model, which can learn the optimal model structure from the data and can flexibly capture the nonlinear dependence between the claim frequency and severity.

Boosting is one of the most successful ensemble learning methods, which combines a large number of weak prediction models (weak learners) in an additive form to enhance prediction performance. The seminal work is Freund and Schapire [22], which introduce a boosting algorithm named AdaBoost for classification. Breiman et al. [23] and Breiman [24] observe an intrinsic connection between the AdaBoost algorithm and the functional gradient descent algorithm. Friedman, Hastie, Tibshirani, et al. [25] reveal another important fact that the AdaBoost and other boosting algorithms are additive models, i.e., an additive combination of weak learners. Then, they propose a general boosting algorithm named gradient boosting for both of classification and regression. The algorithm can be viewed as an estimation method for an additive model that combines weak learners. From this new perspective, many boosting regression models are developed. They are different in forms when different loss functions, weak learners or optimization schemes are used. Friedman, Hastie, and Tibshirani [26] and Friedman [27, 28] develop boosting regression models with the least-squares, least absolute deviation and Huber loss functions. Ridgeway [29, 30] propose the boosting Poisson regression and boosting proportional hazards regression models. Kriegler and Berk [31] introduce the boosting quantile regression model. In actuarial literature, Noll, Salzmann, and Wuthrich [32] show that the boosting Poisson regression model performs better than the GLM model in predicting the claim frequency. Yang, Qian, and Zou [33] develop a gradient boosting Tweedie compound Poisson model, where they use a profile likelihood approach to estimate the index and dispersion parameters. They show that the model makes more accurate premium prediction than GLM and GAM Tweedie compound Poisson models. In order to cope with extremely unbalanced zero-inflated data, Zhou, Yang, and Qian [34] introduce a gradient boosting zero-inflated Tweedie compound Poisson model by using a similar method. In fact, the method that combines the gradient boosting algorithm and profile likelihood approach can be used to develop any gradient boosting exponential family regression models. Sigrist and Hirnschall [35] apply the method to develop a gradient boosting Tobit model for predicting defaults on loans made to Swiss small and medium-sized companies. They show that the model outperforms other state-of-the-art models in predictive performance.

In this paper, we apply the method to develop a gradient boosting frequency-severity model (D-FSBoost). We illustrate the model with a Poisson distribution for modeling the claim frequency and with a gamma distribution for modeling the claim severity. We use the profile likelihood approach to estimate the dispersion parameter in the gamma distribution. The gradient boosting frequency-severity model with other exponential family distributions for modeling the claim frequency and severity can be developed in the same manner. Following Gschlößl and Czado [15], Frees, Gao, and Rosenberg [16], Erhardt and Czado [17], Shi, Feng, and Ivantsova [18] and Garrido, Genest, and Schulz [19], we capture the dependence between the claim frequency and severity by treating the claim frequency as a predictor in the regression model for the average claim severity. Since the gradient boosting gamma regression model can learn the optimal model structure from the data, the D-FSBoost model can fully capture the nonlinear dependence between the claim frequency and severity. The D-FSBoost model inherits all advantages of boosting models, such as the data-driven model structure, high prediction accuracy, automatic feature selection and high capacities of avoiding overfitting problems, etc. In a simulation study, we demonstrate that the D-FSBoost model can flexibly capture the nonlinear relation between the claim frequency (severity) and predictors and complex and higher order interactions among predictors and can fully capture the nonlinear dependence between the claim frequency and severity. We compare the D-FSBoost model with GLM and GAM frequency-severity models and show that the D-FSBoost model can make more accurate prediction in claim frequency and severity distributions. We apply the D-FSBoost model to analyze a French auto insurance claim data. We provide further evidence on the dependence between the claim frequency and severity and indicate that the frequency-severity model can be significantly improved by taking the claim frequency as a predictor in the regression model for the average claim severity. We also show that the D-FSBoost model is superior to other state-of-the-art models in prediction of pure premium.

The rest of this paper is organized as follows. In section 2, we review the gradient boosting algorithm and introduce the D-FSBoost model. In section 3, we show high prediction accuracy of the model in a simulation study. Finally, in section 4, we apply the model to analyze a French auto insurance claim data.

## Stochastic gradient boosting frequency-severity model

In this section, we introduce the stochastic gradient boosting algorithm. Then, we show the implementation of the D-FSBoost model.

### Stochastic gradient boosting

In this subsection, we briefly review the stochastic gradient boosting algorithm in Friedman [28]. Denote by **x** = (*x*_1_, …, *x*_*p*_) the set of predictors and *y* the response variable. Given a training sample ${\left\{y_{i},x_{i}\right\}}_{i=1}^{d}$ and a loss function Ψ(*y*, *f*(**x**)), the algorithm estimates the optimal prediction function $\hat{f}\left(x\right)$ by minimizing loss over the training sample,

$$\begin{array}{c} \hat{f}\left(x\right)=\text{arg}\;\underset{f(x)}{\text{min}}\sum_{i=1}^{d}\Psi \left(y_{i},f\left(x_{i}\right)\right), \end{array}$$ (1)

where *f*(**x**) is constrained to a form of a sum of weak learners as

$$\begin{array}{c} f\left(x\right)=h\left(x;a_{0}\right)+\sum_{m=1}^{M}\beta_{m}h\left(x;a_{m}\right), \end{array}$$ (2)

where *h*(**x**; **a**_*m*_) is a weak learner with a parameter vector **a**_*m*_, $\beta_{m}\in R$ is an expansion coefficient, *M* is the number of weak learners.

The algorithm estimates the function $\hat{f}\left(x\right)$ in a forward stagewise manner. Let the constant *f*_0_(**x**) be an initial estimate of $\hat{f}\left(x\right)$ as

$$\begin{array}{c} f_{0}\left(x\right)=h\left(x;a_{0}\right)=\text{arg}\;\underset{\rho }{\text{min}}\sum_{i=1}^{d}\Psi \left(y_{i},\rho \right). \end{array}$$ (3)

Denote by *f*_*m*−1_(***x***) the estimate of $\hat{f}\left(x\right)$ at the (*m* − 1)^th^ step. Then, at the *m*^th^ step, the algorithm randomly selects a subsample of size $\tilde{d}<d$, ${\left\{{\tilde{y}}_{i},{\tilde{x}}_{i}\right\}}_{1}^{\tilde{d}}$, computes the negative gradient

$$\begin{array}{c} {\tilde{z}}_{i}=-{\frac{\partial \Psi ({\tilde{y}}_{i},f\left({\tilde{x}}_{i}\right))}{\partial f({\tilde{x}}_{i})}|}_{f\left({\tilde{x}}_{i}\right)=f_{m-1}\left({\tilde{x}}_{i}\right)}, \end{array}$$ (4)

and then fits the weak learner *h*(**x**; **a**_*m*_) by minimizing the following least square sum

$$\begin{array}{c} a_{m}=\text{arg}\;\underset{a}{\text{min}}\sum_{i=1}^{\tilde{d}}{\left({\tilde{z}}_{i}-h\left({\tilde{x}}_{i};a\right)\right)}^{2}. \end{array}$$ (5)

The optimal value of *β*_*m*_ is determined by

$$\begin{array}{c} \beta_{m}=\text{arg}\;\underset{\beta }{\text{min}}\sum_{i=1}^{\tilde{d}}\Psi \left({\tilde{y}}_{i},f_{m-1}\left({\tilde{x}}_{i}\right)+\beta h\left({\tilde{x}}_{i};a_{m}\right)\right). \end{array}$$ (6)

Then, the current estimate of $\hat{f}\left(x\right)$ is updated as

$$\begin{array}{c} f_{m}\left(x\right)=f_{m-1}\left(x\right)+\nu \beta_{m}h\left(x;a_{m}\right), \end{array}$$ (7)

where 0 < *ν* ≤ 1 is the shrinkage factor that controls the learning rate. Friedman [27] points out that small *ν* reduces overfitting and enhances predictive performance.

The algorithm reduces to a standard gradient boosting algorithm when the full sample is used at each iteration in place of the randomly selected subsample. Friedman [28] shows that the stochastic gradient boosting algorithm has a faster computation speed and higher prediction accuracy.

### The D-FSBoost model

In this subsection, we introduce the dependent frequency-severity model. Then, we estimate mean parameters by using the stochastic gradient boosting algorithm.

In the frequency-severity model, we model the claim frequency *N* with a Poisson distribution with the parameter λ > 0,

$$\begin{array}{c} f_{N}\left(n|\lambda \right)=\frac{\lambda^{n}}{n!}e^{-\lambda }\;\text{for}\;n=0,1,2,\ldots . \end{array}$$ (8)

For *N* > 0, denote by

$$\begin{array}{c} \tilde{Y}=\frac{Y_{1}+\ldots +Y_{N}}{N} \end{array}$$ (9)

the average claim severity, where *Y*_*j*_ is the *j*^th^ claim amount. We model the average claim severity $\tilde{Y}$ conditional on *N* via a gamma distribution with parameters *μ*_*N*_ > 0 and *δ* > 0

$$\begin{array}{c} f_{\tilde{Y}|N}\left(s|\mu_{N},\delta \right)=\frac{1}{s\Gamma (\frac{1}{\delta })}{\left(\frac{s}{\mu_{N}\delta }\right)}^{\frac{1}{\delta }}e^{-\frac{s}{\mu_{N}\delta }}\;\text{for}\;s>0, \end{array}$$ (10)

where we model the dependence between the claim frequency and severity by making the mean parameter *μ*_*N*_ depend on *N*.

Denote by ***x*** the vector of predictors representing characteristics of an individual policy. We assume that the parameters λ and *μ*_*N*_ are determined by the following two regression models:

$$\begin{array}{c} \text{log}\left(\lambda \right)=F_{N}\left(x;\alpha \right)\;\text{and}\;\text{log}\left(\mu_{N}\right)=F_{\tilde{Y}|N}\left(x,N;\beta \right), \end{array}$$ (11)

where log link functions are used, $F_{N}:R^{p}\to R$ and $F_{\tilde{Y}|N}:R^{p}\times N\to R$ are two regression functions, and ***α*** and ***β*** denote the vector of parameters for *F*_*N*_ and $F_{\tilde{Y}|N}$, respectively. The functions *F*_*N*_ and $F_{\tilde{Y}|N}$ are restricted to linear and additive forms in GLM and GAM models, respectively. In our model, *F*_*N*_ and $F_{\tilde{Y}|N}$ are ensembles of weak learners.

For the time being, we assume that the dispersion parameter *δ* is given. We will estimate *δ* later. Then, we apply the stochastic gradient boosting algorithm to estimate the functions *F*_*N*_ and $F_{\tilde{Y}|N}$.

Denote by {*n*_*i*_, *s*_*i*_, ***x***_*i*_} the claim frequency, the average claim severity and the vector of predictors for the *i*^th^ policy, respectively. We consider *θ* independent insurance policies. Then, we have the log-likelihood function as follows:

$$\begin{array}{c} \begin{array}{cc} \ell (\alpha ,\beta ,\delta |{\left\{n_{i},s_{i},x_{i}\right\}}_{i=1}^{\theta }) & =\underset{l_{1}\left(\alpha \right)}{\underset{︸}{\sum_{i=1}^{\theta }\text{log}\frac{e^{n_{i}F_{N}\left(x_{i};\alpha \right)}}{n_{i}!}e^{-e^{F_{N}\left(x_{i};\alpha \right)}}}}\\ & \;+\underset{l_{2}\left(\beta ,\delta \right)}{\underset{︸}{\sum_{i=1}^{\theta }\text{log}\frac{1}{s_{i}\Gamma (\frac{1}{\delta })}{\left(\frac{s_{i}}{\delta e^{F_{\tilde{Y}|N}\left(x_{i},n_{i};\beta \right)}}\right)}^{\frac{1}{\delta }}e^{-\frac{s_{i}}{\delta e^{F_{\tilde{Y}|N}\left(x_{i},n_{i};\beta \right)}}}}} \end{array}. \end{array}$$ (12)

Since maximizing the above log-likelihood function is equivalent to maximizing *l*_1_(***α***) and *l*_2_(***β***, *δ*), respectively, we use negative log-likelihood functions −*l*_1_(***α***) and −*l*_2_(***β***, *δ*) as loss functions and estimate the functions *F*_*N*_(***x***;***α***) and $F_{\tilde{Y}|N}\left(x,N;\beta \right)$ by minimizing loss over the sample ${\left\{n_{i},s_{i},x_{i}\right\}}_{i=1}^{\theta }$,

$$\begin{array}{c} \hat{f}\left(x\right)=\text{arg}\;\underset{f(x)}{\text{min}}\sum_{i=1}^{\theta }\Psi_{1}\left(n_{i},f\left(x_{i}\right)\right)\;\text{and}\;\hat{g}\left(x,N\right)=\text{arg}\;\underset{g(x,N)}{\text{min}}\sum_{i=1}^{\theta }\Psi_{2}\left(s_{i},g\left(x_{i},n_{i}\right)\right), \end{array}$$ (13)

where

$$\begin{array}{c} \left\{ \begin{array}{cc} \Psi_{1}\left(n_{i},f\left(x_{i}\right)\right) & =-\text{log}\frac{e^{n_{i}f\left(x_{i}\right)}}{n_{i}!}e^{-e^{f(x_{i})}}\\ \Psi_{2}\left(s_{i},g\left(x_{i},n_{i}\right)\right) & =-\text{log}\frac{1}{s_{i}\Gamma (\frac{1}{\delta })}{\left(\frac{s_{i}}{\delta e^{g(x_{i},n_{i})}}\right)}^{\frac{1}{\delta }}e^{-\frac{s_{i}}{\delta e^{g(x_{i},n_{i})}}} \end{array},\right. \end{array}$$ (14)

and the functions *f*(**x**) and *g*(**x**, *N*) are confined to the form of a sum of weak learners as (2).

Then, the gradient boosting algorithm estimate $\hat{f}\left(x\right)$ and $\hat{g}\left(x,N\right)$ in a forward stagewise manner. The initial estimates are computed as

$$\begin{array}{c} \left\{ \begin{array}{c} f_{0}\left(x\right)=\text{arg}\;\underset{\rho }{\text{min}}\sum_{i=1}^{\theta }\Psi_{1}\left(n_{i},\rho \right)\\ g_{0}\left(x,N\right)=\text{arg}\;\underset{\rho }{\text{min}}\sum_{i=1}^{\theta }\Psi_{2}\left(s_{i},\rho \right) \end{array}.\right. \end{array}$$ (15)

Denote by *f*_*m*−1_(**x**) and *g*_*m*−1_(**x**, *N*) the estimates of $\hat{f}\left(x\right)$ and $\hat{g}\left(x,N\right)$ at the (*m* − 1)^th^ step, respectively. At the *m*^th^ step, the algorithm randomly selects a subsample of size $\tilde{\theta }<\theta$, ${\left\{{\tilde{n}}_{i},{\tilde{s}}_{i},{\tilde{x}}_{i}\right\}}_{i=1}^{\tilde{\theta }}$, and computes the negative gradient

$$\begin{array}{c} \left\{ \begin{array}{cc} {\tilde{z}}_{i}^{f} & ={\tilde{n}}_{i}-e^{f_{m-1}\left({\tilde{x}}_{i}\right)}\\ {\tilde{z}}_{i}^{g} & =\frac{{\tilde{s}}_{i}e^{-g_{m-1}\left({\tilde{x}}_{i},{\tilde{n}}_{i}\right)}-1}{\delta } \end{array}.\right. \end{array}$$ (16)

Then, the algorithm fits weak learners $h^{f}\left(x;a_{m}^{f}\right)$ and $h^{g}\left(x,N;a_{m}^{g}\right)$ by minimizing the following least square sums,

$$\begin{array}{c} \left\{ \begin{array}{cc} a_{m}^{f} & =\text{arg}\;\underset{a}{\text{min}}\sum_{i=1}^{\tilde{\theta }}{\left({\tilde{z}}_{i}^{f}-h^{f}\left({\tilde{x}}_{i};a\right)\right)}^{2}\\ a_{m}^{g} & =\text{arg}\;\underset{a}{\text{min}}\sum_{i=1}^{\tilde{\theta }}{\left({\tilde{z}}_{i}^{g}-h^{g}\left({\tilde{x}}_{i},{\tilde{n}}_{i};a\right)\right)}^{2} \end{array}.\right. \end{array}$$ (17)

We use K-terminal node regression trees as weak learners, i.e.,

$$\begin{array}{c} \left\{ \begin{array}{c} h^{f}\left(x;a_{m}^{f}\right)=\sum_{k=1}^{K}{\hat{n}}_{k}1_{\left\{x\in U_{k,m}\right\}}\\ h^{g}\left(x,N;a_{m}^{g}\right)=\sum_{k=1}^{K}{\hat{s}}_{k}1_{\left\{\left(x,N\right)\in V_{k,m}\right\}} \end{array},\right. \end{array}$$ (18)

where

$$\begin{array}{c} \left\{ \begin{array}{cc} {\hat{n}}_{k} & =\frac{\sum_{i=1}^{\tilde{\theta }}{\tilde{n}}_{i}1_{\left\{{\tilde{x}}_{i}\in U_{k,m}\right\}}}{\sum_{i=1}^{\tilde{\theta }}1_{\left\{{\tilde{x}}_{i}\in U_{k,m}\right\}}}\\ {\hat{s}}_{k} & =\frac{\sum_{i=1}^{\tilde{\theta }}{\tilde{s}}_{i}1_{\left\{\left({\tilde{x}}_{i},{\tilde{n}}_{i}\right)\in V_{k,m}\right\}}}{\sum_{i=1}^{\tilde{\theta }}1_{\left\{\left({\tilde{x}}_{i},{\tilde{n}}_{i}\right)\in V_{k,m}\right\}}} \end{array},\right. \end{array}$$ (19)

and ${\left\{U_{k,m}\right\}}_{k=1}^{K}$ and ${\left\{V_{k,m}\right\}}_{k=1}^{K}$ are disjoint regions of **x** and (**x**, *N*) spaces, respectively, which represent terminal nodes of regression trees. In this case, the parameters $a_{m}^{f}$ and $a_{m}^{g}$ are splitting variables and split points of regression trees, which determine the regions ${\left\{U_{k,m}\right\}}_{k=1}^{K}$ and ${\left\{V_{k,m}\right\}}_{k=1}^{K}$. The optimization problem (17) is solved by a greedy algorithm with a least squared splitting criterion (Friedman [27]).

Once the weak learners $h^{f}\left(x;a_{m}^{f}\right)$ and $h^{g}\left(x,N;a_{m}^{g}\right)$ are obtained, the optimal expansion coefficients $\beta_{m}^{f}$ and $\beta_{m}^{g}$ are solved by

$$\begin{array}{c} \left\{ \begin{array}{cc} \beta_{m}^{f} & =\text{arg}\;\underset{\beta }{\text{min}}\sum_{i=1}^{\tilde{\theta }}\Psi_{1}({\tilde{n}}_{i},f_{m-1}\left({\tilde{x}}_{i}\right)+\beta \sum_{k=1}^{K}{\hat{n}}_{k}1_{\left\{{\tilde{x}}_{i}\in U_{k,m}\right\}})\\ \beta_{m}^{g} & =\text{arg}\;\underset{\beta }{\text{min}}\sum_{i=1}^{\tilde{\theta }}\Psi_{2}({\tilde{s}}_{i},g_{m-1}\left({\tilde{x}}_{i},{\tilde{n}}_{i}\right)+\beta \sum_{k=1}^{K}{\hat{s}}_{k}1_{\left\{\left({\tilde{x}}_{i},{\tilde{n}}_{i}\right)\in V_{k,m}\right\}}) \end{array}.\right. \end{array}$$ (20)

We can obtain the better estimation of $\hat{f}\left(x\right)$ and $\hat{g}\left(x,N\right)$ by replacing a single expansion coefficient $\beta_{m}^{f}$ ($\beta_{m}^{g}$) with the optimal coefficient $\gamma_{k,m}^{f}$ ($\gamma_{k,m}^{g}$), *k* = 1, …, *K* for each region *U*_*k*,*m*_ (*V*_*k*,*m*_), *k* = 1, …, *K*. The optimal coefficients $\gamma_{k,m}^{f}$ ($\gamma_{k,m}^{g}$), *k* = 1, …, *K* are solved by

$$\begin{array}{c} \left\{ \begin{array}{cc} \gamma_{k,m}^{f} & =\text{arg}\;\underset{\gamma }{\text{min}}\sum_{{\tilde{x}}_{i}\in U_{k,m}}\Psi_{1}\left({\tilde{n}}_{i},f_{m-1}\left({\tilde{x}}_{i}\right)+\gamma \right)\\ \gamma_{k,m}^{g} & =\text{arg}\;\underset{\gamma }{\text{min}}\sum_{\left({\tilde{x}}_{i},{\tilde{n}}_{i}\right)\in V_{k,m}}\Psi_{2}\left({\tilde{s}}_{i},g_{m-1}\left({\tilde{x}}_{i},{\tilde{n}}_{i}\right)+\gamma \right) \end{array}.\right. \end{array}$$ (21)

We have explicit solutions as follows:

$$\begin{array}{c} \left\{ \begin{array}{cc} \gamma_{k,m}^{f} & =\text{log}(\frac{\sum_{i=1}^{\tilde{\theta }}{\tilde{n}}_{i}1_{\left\{{\tilde{x}}_{i}\in U_{k,m}\right\}}}{\sum_{i=1}^{\tilde{\theta }}e^{f_{m-1}\left({\tilde{x}}_{i}\right)}1_{\left\{{\tilde{x}}_{i}\in U_{k,m}\right\}}})\\ \gamma_{k,m}^{g} & =\text{log}(\frac{\sum_{i=1}^{\tilde{\theta }}{\tilde{s}}_{i}e^{-g_{m-1}\left({\tilde{x}}_{i},{\tilde{n}}_{i}\right)}1_{\left\{\left({\tilde{x}}_{i},{\tilde{n}}_{i}\right)\in V_{k,m}\right\}}}{\sum_{i=1}^{\tilde{\theta }}1_{\left\{\left({\tilde{x}}_{i},{\tilde{n}}_{i}\right)\in V_{k,m}\right\}}}) \end{array}.\right. \end{array}$$ (22)

Then, the estimates of $\hat{f}\left(x\right)$ and $\hat{g}\left(x,N\right)$ are updated as

$$\begin{array}{c} \left\{ \begin{array}{cc} f_{m}\left(x\right) & =f_{m-1}\left(x\right)+\nu \sum_{k=1}^{K}\gamma_{k,m}^{f}1_{x\in U_{k,m}}\\ g_{m}\left(x,N\right) & =g_{m-1}\left(x,N\right)+\nu \sum_{k=1}^{K}\gamma_{k,m}^{g}1_{\left(x,N\right)\in V_{k,m}} \end{array},\right. \end{array}$$ (23)

where we set *ν* = 0.03 in our implementation.

The procedures are repeated *M* times. Then, we obtain *f*_*M*_(**x**) and *g*_*M*_(**x**, *N*) as the final estimates.

The D-FSBoost algorithm is summarized as follows:

**The D-FSBoost Algorithm**

1. Initialize *f*_0_(**x**) and *g*_0_(**x**, *N*),

$$\begin{array}{c} \left\{ \begin{array}{c} f_{0}\left(x\right)=\text{arg}\;\underset{\rho }{\text{min}}\sum_{i=1}^{\theta }\Psi_{1}\left(n_{i},\rho \right)\\ g_{0}\left(x,N\right)=\text{arg}\;\underset{\rho }{\text{min}}\sum_{i=1}^{\theta }\Psi_{2}\left(s_{i},\rho \right) \end{array}.\right. \end{array}$$ (24)

2. For *m* = 1 to *M* do

1. Generate a random subsample ${\left\{{\tilde{n}}_{i},{\tilde{s}}_{i},{\tilde{x}}_{i}\right\}}_{i=1}^{\tilde{\theta }}$.
2. Compute the negative gradient $\left({\tilde{z}}_{1}^{f},\ldots ,{\tilde{z}}_{\tilde{\theta }}^{f}\right)$ and $\left({\tilde{z}}_{1}^{g},\ldots ,{\tilde{z}}_{\tilde{\theta }}^{g}\right)$,
  $$\begin{array}{c} \left\{ \begin{array}{cc} {\tilde{z}}_{i}^{f} & ={\tilde{n}}_{i}-e^{f_{m-1}\left({\tilde{x}}_{i}\right)}\\ {\tilde{z}}_{i}^{g} & =\frac{{\tilde{s}}_{i}e^{-g_{m-1}\left({\tilde{x}}_{i},{\tilde{n}}_{i}\right)}-1}{\delta } \end{array},\;i=1,\ldots ,\tilde{\theta }.\right. \end{array}$$ (25)
3. K-terminal node regression trees fit two datasets ${\left\{{\tilde{z}}_{i}^{f},{\tilde{x}}_{i}\right\}}_{i=1}^{\tilde{\theta }}$ and ${\left\{{\tilde{z}}_{i}^{g},\left({\tilde{x}}_{i},{\tilde{n}}_{i}\right)\right\}}_{i=1}^{\tilde{\theta }}$ with a least squared splitting criterion and obtain the regions ${\left\{U_{k,m}\right\}}_{k=1}^{K}$ and ${\left\{V_{k,m}\right\}}_{k=1}^{K}$.
4. Compute the optimal coefficient for each region *U*_*k*,*m*_ (*V*_*k*,*m*_), *k* = 1, …, *K*,
  $$\begin{array}{c} \left\{ \begin{array}{cc} \gamma_{k,m}^{f} & =\text{log}(\frac{\sum_{i=1}^{\tilde{\theta }}{\tilde{n}}_{i}1_{\left\{{\tilde{x}}_{i}\in U_{k,m}\right\}}}{\sum_{i=1}^{\tilde{\theta }}e^{f_{m-1}\left({\tilde{x}}_{i}\right)}1_{\left\{{\tilde{x}}_{i}\in U_{k,m}\right\}}})\\ \gamma_{k,m}^{g} & =\text{log}(\frac{\sum_{i=1}^{\tilde{\theta }}{\tilde{s}}_{i}e^{-g_{m-1}\left({\tilde{x}}_{i},{\tilde{n}}_{i}\right)}1_{\left\{\left({\tilde{x}}_{i},{\tilde{n}}_{i}\right)\in V_{k,m}\right\}}}{\sum_{i=1}^{\tilde{\theta }}1_{\left\{\left({\tilde{x}}_{i},{\tilde{n}}_{i}\right)\in V_{k,m}\right\}}}) \end{array},\;k=1,\ldots ,K.\right. \end{array}$$ (26)
5. Update the estimates of $\hat{f}\left(x\right)$ and $\hat{g}\left(x,N\right)$ as
  $$\begin{array}{c} \left\{ \begin{array}{cc} f_{m}\left(x\right) & =f_{m-1}\left(x\right)+\nu \sum_{k=1}^{K}\gamma_{k,m}^{f}1_{x\in U_{k,m}}\\ g_{m}\left(x,N\right) & =g_{m-1}\left(x,N\right)+\nu \sum_{k=1}^{K}\gamma_{k,m}^{g}1_{\left(x,N\right)\in V_{k,m}} \end{array}.\right. \end{array}$$ (27)

end

3. Return *f*_*M*_(**x**) and *g*_*M*_(**x**, *N*).

### Estimating *δ* and choice of tuning parameters

We estimate the dispersion parameter *δ* using the profile likelihood approach. The D-FSBoost algorithm determines the value of ***β*** for each fixed *δ*. Denote by ***β***_*δ*_ the estimated value of ***β***. Then, the profile log-likelihood function for *δ* is given by

$$\begin{array}{c} \tilde{l}\left(\delta \right)=l_{2}\left(\beta_{\delta },\delta \right). \end{array}$$ (28)

The value of the dispersion parameter *δ* is obtained by maximizing the profile log-likelihood function $\tilde{l}\left(\delta \right)$:

$$\begin{array}{c} \hat{\delta }=\arg\max_{\delta }\;\tilde{l}\left(\delta \right). \end{array}$$ (29)

To reduce computations, we calculate $\hat{\delta }$ by doing a simple grid search over *S* grid points {*δ*_1_, …, *δ*_*S*_}, i.e.,

$$\begin{array}{c} \hat{\delta }=\arg\max_{\delta \in \{\delta_{1},\ldots ,\delta_{S}\}}\tilde{l}\left(\delta \right). \end{array}$$ (30)

In the implementation, we need select tuning parameters, including the size of trees *K* and the number of trees *M*. The value of *K* controls the degree of the interaction among the predictors ***x*** or (*N*, ***x***). The appropriate value of *M* avoids over-fitting and improves out-of-sample prediction accuracy. We determine the parameters (*K*, *M*) via the cross-validation method. The *k*−fold cross-validation method splits the data into *k* equal-sized folds. Let *κ*(*i*):{1, …, *θ*} → {1, …, *k*} be an index function that indicates the fold to which the *i*^th^ observation is allocated by randomization. We calculate loss of the *j*^th^ fold data by using functions estimated by the remaining *k* − 1 folds. We repeat this procedure for *j* = 1, …, *k*. Denote by $\left({\hat{f}}_{-j}\left(x,K,M\right),{\hat{g}}_{-j}\left(x,N,K,M\right)\right)$ the functions estimated with the *j*^th^ fold data removed and with the parameters (*K*, *M*). Then, the cross-validation estimate of loss is

$$\begin{array}{c} \text{CV}\left(K,M\right)=\sum_{i=1}^{\theta }\left(\Psi_{1}\left(n_{i},f_{-\kappa (i)}\left(x_{i},K,M\right)\right)+\Psi_{2}\left(s_{i},g_{-\kappa (i)}\left(x_{i},n_{i},K,M\right)\right)\right). \end{array}$$ (31)

The optimal (*K*, *M*) is obtained by minimizing the cross-validation estimate of loss

$$\begin{array}{c} \left(\hat{K},\hat{M}\right)=\text{arg}\;\underset{K,M}{\text{min}}\text{CV}\left(K,M\right). \end{array}$$ (32)

Then, we use $\left(\hat{K},\hat{M}\right)$ in the D-FSBoost algorithm and finish all estimates.

## Simulation study

In this section, we compare the D-FSBoost model with GLM and GAM frequency-severity models in two simulation experiments. We consider the models in the cases that the frequency and severity are independent and dependent. Denote by D-FSBoost, D-GAM and D-GLM the three models in the dependent case and by FSBoost, GAM and GLM the three models in the independent case. We compare the models in prediction accuracy of the claim frequency and severity distributions. We also investigate the impact of the value of *δ* on estimating $F_{\tilde{Y}|N}\left(x,N;\beta \right)$ in the D-FSBoost model.

In simulation studies, we use one set of samples for training and another one for testing. Denote by ${\left\{{\hat{n}}_{i},{\hat{s}}_{i},{\hat{x}}_{i}\right\}}_{i=1}^{\hat{\theta }}$ the testing sample with known true functions or parameters $\left\{F_{N}\left(x;\alpha \right),F_{\tilde{Y}|N}\left(x,N;\beta \right),\delta \right\}$. Let $\left\{\hat{f}\left(x\right),\hat{g}\left(x,N\right),\hat{\delta }\right\}$ be the functions or parameters estimated by the model. We use the out-of-sample loss and parameter estimation errors to measure prediction accuracy of the models. Table 1 shows the specific performance measures. In the FSBoost and D-FSBoost models, we use the five-fold cross-validation method to select parameters (*K*, *M*) among the combinations of *K* ∈ {1, 2, 3, 4, 5} and *M* ∈ {100, 200, 300, 400, 500} and search the optimal *δ* among 21 equally spaced values {1, 1.1, …, 3}.

**Table 1:** Performance measures.

Measure	Description	Formula
Frequency Loss	Out-of-sample loss for the claim frequency	$\sum_{i=1}^{\hat{\theta }}\Psi_{1}\left({\hat{n}}_{i},\hat{f}\left({\hat{x}}_{i}\right)\right)$
Severity Loss	Out-of-sample loss for the claim severity	$\sum_{i=1}^{\hat{\theta }}\Psi_{2}\left({\hat{s}}_{i},\hat{g}\left({\hat{x}}_{i},{\hat{n}}_{i}\right)\right)$
Frequency Error	Average relative error of *F*_*N*_(*x*_*i*_;***α***)	$\frac{1}{\hat{\theta }}\sum_{i=1}^{\hat{\theta }}\frac{|\hat{f}\left({\hat{x}}_{i}\right)-F_{N}\left({\hat{x}}_{i};\alpha \right)|}{F_{N}\left({\hat{x}}_{i};\alpha \right)}$
Severity Error	Average relative error of $F_{\tilde{Y}|N}\left(x,N;\beta \right)$	$\frac{1}{\hat{\theta }}\sum_{i=1}^{\hat{\theta }}\frac{|\hat{g}\left({\hat{x}}_{i},{\hat{n}}_{i}\right)-F_{\tilde{Y}|N}\left({\hat{x}}_{i},{\hat{n}}_{i};\beta \right)|}{F_{\tilde{Y}|N}\left({\hat{x}}_{i},{\hat{n}}_{i};\beta \right)}$
*δ* Estimation Error	Relative error of *δ*	$\frac{|\hat{\delta }-\delta |}{\delta }$

### Simple case

In this subsection, we demonstrate that the D-FSBoost model can capture the nonlinear relation between the claim frequency (severity) and predictors, complex interactions among predictors, and the nonlinear dependence between the claim frequency and severity. The sample ${\left\{n_{i},s_{i},x_{i}\right\}}_{i=1}^{\theta }$ is generated using the following specifications,

$$\begin{array}{c} n_{i}∼\text{Poi}\left(\lambda_{i}\right),\;s_{i}∼\text{Gamma}\left(\mu_{n_{i}},\delta \right),\;x_{ij}∼\text{Unif}\left(0,1\right),\;i=1,\ldots ,\theta ,\;j=1,\ldots ,4, \end{array}$$ (33)

where λ_*i*_ = exp(*F*_1_(*x*_*i*1_, *x*_*i*2_)), $\mu_{n_{i}}=\text{exp}\left(F_{2}\left(x_{i3},x_{i4},n_{i}\right)\right)$, *δ* = 2, and

$$\begin{array}{c} \left\{ \begin{array}{cc} F_{1}\left(x_{i1},x_{i2}\right) & =\frac{\pi }{15}\left(3x_{i1}^{2}+2{\left(1-x_{i2}\right)}^{2}+10x_{i1}x_{i2}\right)\\ F_{2}\left(x_{i3},x_{i4},n_{i}\right) & =\ln\left(n_{i}+3\right)e^{x_{i3}^{2}-2{\left(1-x_{i4}\right)}^{2}}+\ln\left(n_{i}+5\right)e^{\frac{1}{2}x_{i3}x_{i4}} \end{array}.\right. \end{array}$$ (34)

We generate a sample of size 10000 for training and another one of size 10000 for testing. The out-of-sample loss and parameter estimation errors on the testing sample are shown in Table 2, which are averaged over 20 independent replications. Since the independent and dependent models share the same claim frequency model, we only list the claim frequency result for the dependent models. We can find that dependent models perform better than independent ones. In dependent models, the D-FSBoost model has the best performance in terms of the smallest out-of-sample loss and parameter estimation errors.

**Table 2:** Out-of-sample loss and parameter estimation errors.

	Frequency Loss	Severity Loss	Frequency Error	Severity Error	*δ* Estimation Error
GLM	-	55005.72 (3731.04)	-	1.3651 (0.0893)	6.9003 (3.9905)
D-GLM	18450.51 (89.25)	46753.29 (322.34)	0.1516 (0.0030)	0.4802 (0.0181)	0.6908 (0.1391)
GAM	-	50678.64 (1409.73)	-	1.2669 (0.0740)	2.8192 (1.0205)
D-GAM	18384.86 (84.01)	46087.76 (218.75)	0.1449 (0.0023)	0.3928 (0.0139)	0.3200 (0.0539)
FSBoost	-	46886.52 (166.85)	-	0.9186 (0.0315)	0.2075 (0.0373)
D-FSBoost	18114.45 (74.25)	45541.16 (176.06)	0.0702 (0.0153)	0.1217 (0.0083)	0.0150 (0.0235)

**Fig 1:**
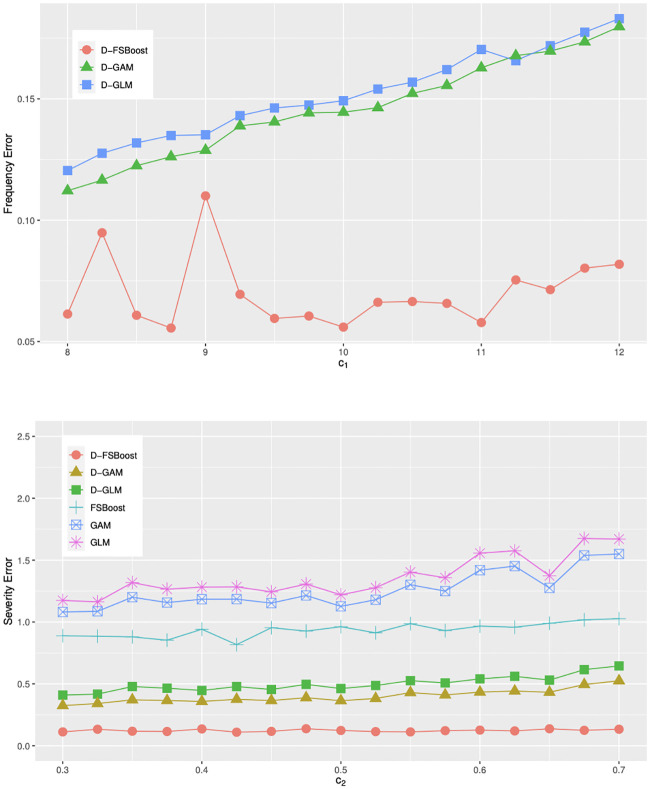
Parameter estimation errors w.r.t. *c*_1_ and *c*_2_.

In contrast to the GLM, D-GLM, GAM and D-GAM models, the FSBoost and D-FSBoost models can capture complex interactions. Denote by *c*_1_ and *c*_2_ the coefficients of cross-product terms *x*_*i*1_ *x*_*i*2_ and *x*_*i*3_ *x*_*i*4_, respectively. In Fig 1, we change *c*_1_ from 8 to 12 and *c*_2_ from 0.3 to 0.7 to increase effects of interaction terms. We can find that the FSBoost and D-FSBoost models have more stable predictive performance. In the GLM, D-GLM, GAM and D-GAM models, the parameter estimation errors show an increasing trend since they can’t capture interaction effects. Next, we use values of *x*_*i*3_ and *x*_*i*4_ in the training sample and change all values of *n*_*i*_, *i* = 1, …, 10000, from 0 to 20. For each value of *n*_*i*_, *i* = 1, …, 10000, we calculate

$$\begin{array}{c} s=\frac{1}{10000}\sum_{i=1}^{10000}\hat{g}\left(x_{i3},x_{i4},n_{i}\right). \end{array}$$ (35)

**Fig 2:**
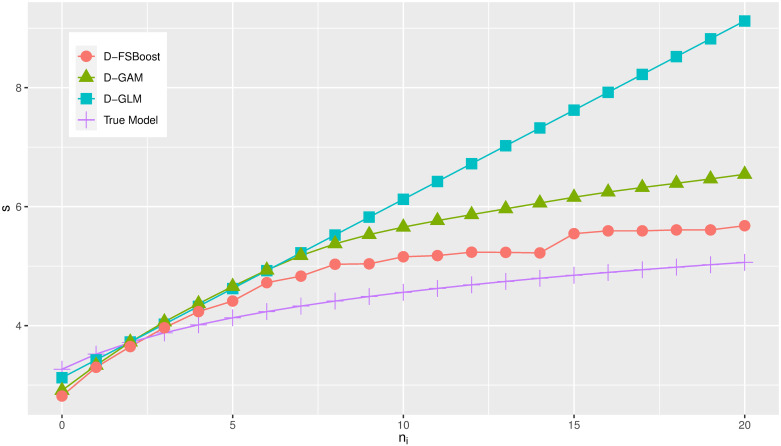
The change of *s* w.r.t. *n*_*i*_.

Then, we show the change of *s* with respect to *n*_*i*_ in Fig 2. The D-GLM model can only measure a linear relation between the claim frequency and severity. Both of the D-GAM and D-FSBoost models can capture the nonlinear dependence between the claim frequency and severity. The D-FSBoost model performs better. The results also confirm that the D-FSBoost model can capture the nonlinear relation between the claim frequency (severity) and predictors.

### Complex case

In this subsection, we demonstrate the D-FSBoost model in a complex simulation experiment. We compare the models on a variety of randomly generated functions by using the “random function generator” in Friedman [27].

The “random function generator” generates a function as a linear expansion of functions ${\left\{g_{k}\right\}}_{k=1}^{20}:$

$$\begin{array}{c} F\left(x\right)=\sum_{k=1}^{20}a_{k}g_{k}\left(z_{k}\right). \end{array}$$ (36)

Each coefficient *a*_*k*_ is generated from a uniform distribution on (0, 1). The variables ***z***_*k*_ is a *m*_*k*_−sized subset of *p*-input variables ***x*** as

$$\begin{array}{c} z_{k}={\left\{x_{\phi (k)}\right\}}_{k=1}^{m_{k}}, \end{array}$$ (37)

where *ϕ*(*k*) is an independent random permutation of integers {1, …, *p*}. The size *m*_*k*_ is randomly selected as min(⌊2.5 + *r*_*k*_⌋, *p*), where *r*_*k*_ is generated from an exponential distribution with mean 2. Then, the expected number of input variables for each *g*_*k*_(***z***_*k*_) is between four and five. Each *g*_*k*_(***z***_*k*_) is an *m*_*k*_−dimensional Gaussian function

$$\begin{array}{c} g_{k}\left(z_{k}\right)=\text{exp}(-\frac{1}{2}{\left(z_{k}-u_{k}\right)}^{T}V_{k}\left(z_{k}-u_{k}\right)), \end{array}$$ (38)

where each mean vector ***u***_*k*_ is generated from the same distribution as ***z***_*k*_. The *m*_*k*_ × *m*_*k*_ covariance matrix *V*_*k*_ is generated by

$$\begin{array}{c} V_{k}=U_{k}D_{k}U_{k}^{T}, \end{array}$$ (39)

where *U*_*k*_ is a random orthonormal matrix, $D_{k}=\text{diag}\left\{d_{1}^{k},\ldots ,d_{m_{k}}^{k}\right\}$, and the square root of each eigenvalue $\sqrt{d_{j}^{k}}$ is generated from a uniform distribution on (*a*, *b*), where the values of *a* and *b* are determined by the distribution of ***z***_*k*_. We set the number of predictors *p* = 10 and generate the data ${\left\{n_{i},s_{i},x_{i},y_{i}\right\}}_{i=1}^{\theta }$ using the following specifications,

$$\begin{array}{c} n_{i}∼\text{Poi}\left(\lambda_{i}\right),\;s_{i}∼\text{Gamma}\left(\mu_{n_{i}},\delta \right),\;x_{i}∼N\left(0,I_{p}\right),\;y_{i}∼N\left(0,I_{p}\right),\;i=1,\ldots ,\theta , \end{array}$$ (40)

where λ_*i*_ = 1.2exp(*F*_1_(*x*_*i*_)), $\mu_{n_{i}}=\text{exp}\left(\text{log}\left(n_{i}+5\right)F_{2}\left(y_{i}\right)\right)$, *δ* = 2, and *F*_1_(***x***_*i*_) and *F*_2_(***y***_*i*_) are the functions generated from the “random function generator”. The eigenvalue limits are *a* = 0.1 and *b* = 2.

**Fig 3:**
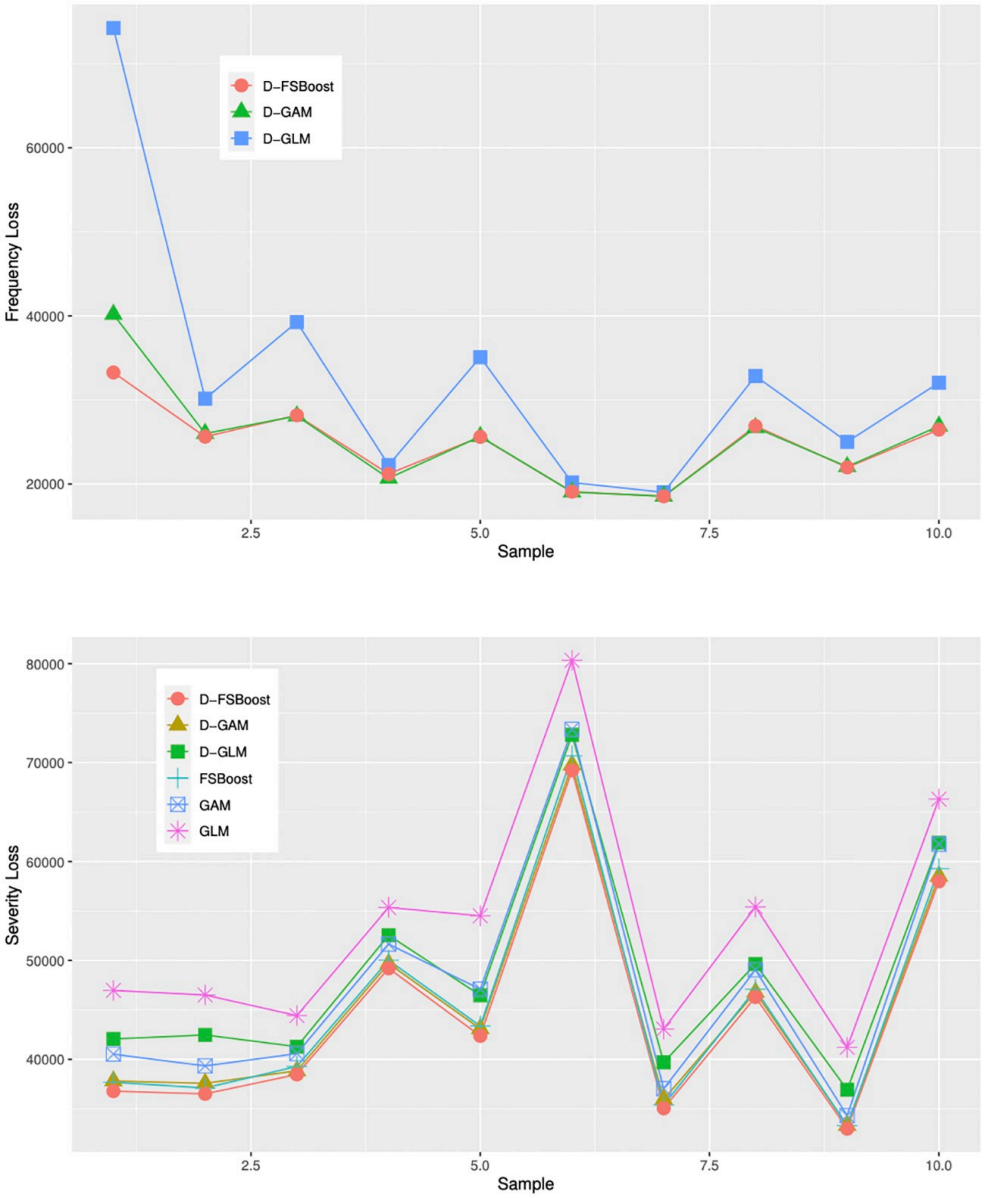
Out-of-sample loss.

We generate 10000 observations for training and another 10000 for testing. Table 3 reports parameter estimation errors on the testing sample, which are averaged over 10 independent replications. Fig 3 shows out-of-sample loss. The results are the same as in the simple case. Dependent models have more accurate prediction than independent models. The D-FSBoost model performs best in predicting the claim frequency and severity distributions.

**Table 3:** Parameter estimation errors.

	Frequency Error	Severity Error	*δ* Estimation Error
GLM	-	4.2459 (1.6405)	6.3718 (2.1039)
D-GLM	0.4529 (0.1768)	2.9406 (0.9037)	2.4299 (0.6622)
GAM	-	1.2852 (0.3275)	1.8402 (0.6285)
D-GAM	0.2175 (0.0500)	0.7398 (0.1398)	0.6570 (0.1870)
FSBoost	-	0.8238 (0.2403)	0.2500 (0.0667)
D-FSBoost	0.2092 (0.0411)	0.4562 (0.0441)	0.0950 (0.0284)

### The impact of the parameter *δ*

**Fig 4:**
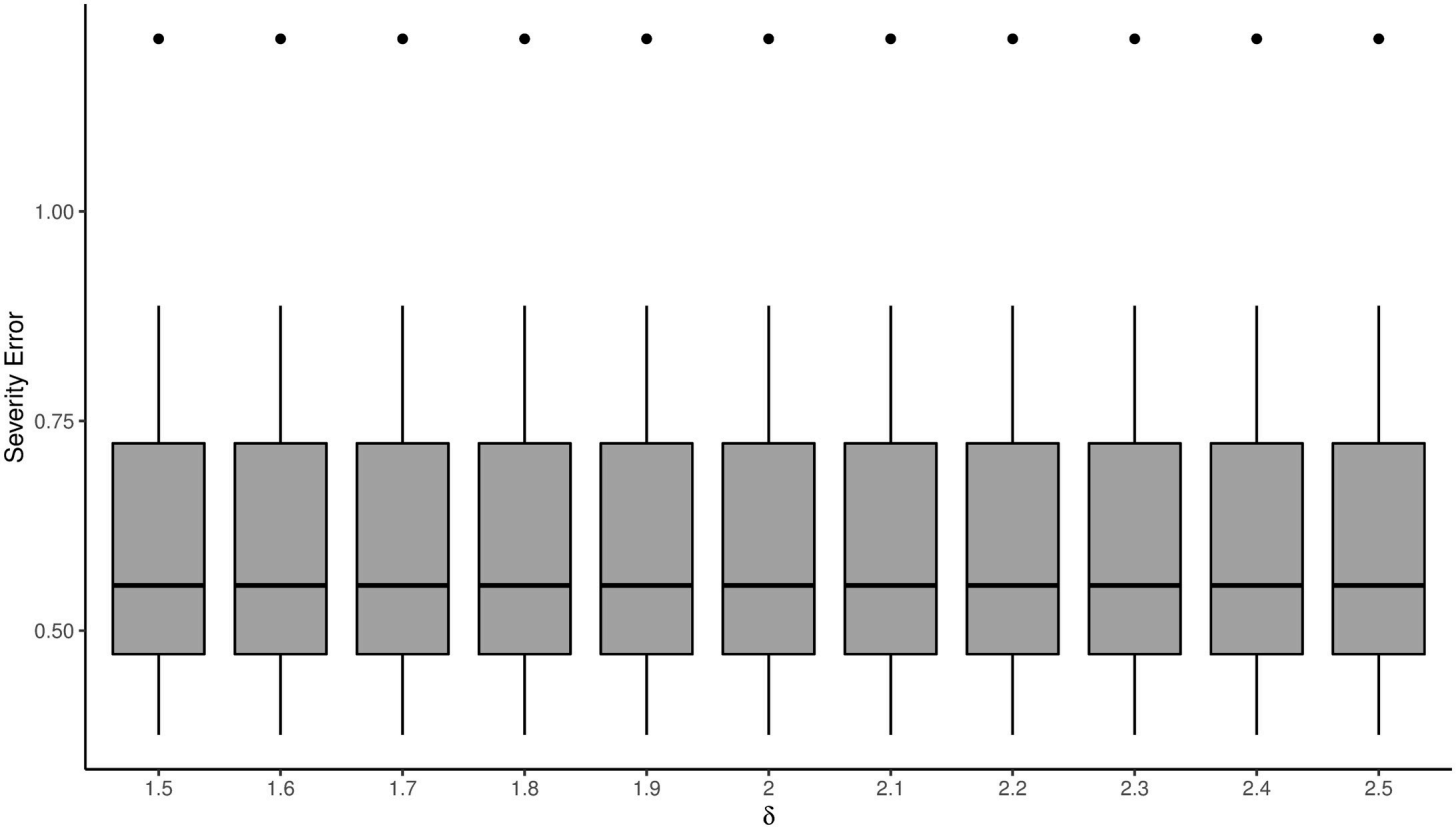
Parameter estimation errors of the D-FSBoost model when varying the value of *δ* from 1.5 to 2.5.

In this subsection, we investigate the impact of the value of *δ* on estimating $F_{\tilde{Y}|N}\left(x,N;\beta \right)$. We generate 20 sets of training samples as in the complex case. Then, we estimate $F_{\tilde{Y}|N}\left(x,N;\beta \right)$ using the D-FSBoost algorithm for each value of *δ* ∈ {1.5, 1.6, …, 2.5}. Fig 4 shows parameter estimation errors. We can see that the value of *δ* has no significant effect on estimation accuracy of $F_{\tilde{Y}|N}\left(x,N;\beta \right)$.

## Application

In this section, we apply the D-FSboost model to analyze a French auto insurance claim data. We compare the models in prediction of the claim frequency and severity distributions. Then, we introduce two important tools, variable importance measures and partial dependence plots, from Friedman [27] to interpret the D-FSBoost model.

### Data

We consider a French motor third-party liability dataset, where the data “freMTPL2freq” and “freMTPL2sev” are in the R package “CASdatasets”. Noll, Salzmann, and Wuthrich [32] use the data “freMTPL2freq” to compare the GLM, regression tree, gradient boosting Poisson model and neural network in predicting the claim frequency. We make the same data preprocess as in Noll, Salzmann, and Wuthrich [32], except deleting records that have positive claim frequency but have no claim severity and also except using different partitions on variables “VehAge” and “DrivAge”. After the data preprocess, the dataset contains 668897 records. The dataset is openly available from S1 Dataset. Table 4 shows variables in the dataset. There are 24944 (3.73%) policies that have positive claim frequency. Table 5 reports the distribution of the claim frequency and average claim severity. There are only several policies in which the claim frequency is larger than 3. The average claim severity shows an increasing trend when the claim frequency changes from 0 to 3. This implies a positive dependence structure between the claim frequency and severity.

**Table 4:** Variables.

Variable	Type	Description
ClaimNb	Numeric	The claim frequency during the exposure period
Exposure	Numeric	The period of exposure for a policy in years
ClaimSev	Numeric	The average claim severity
Area	Categorical	The density value of the city community where the policyholder lives: from “1” for rural area to “6” for urban centre (1-6)
VehPower	Categorical	The power of the car (6 classes)
VehAge	Categorical	The vehicle age in years ((0,1], (1,4], (4,10], (10,∞))
DrivAge	Categorical	The driver age in years ([18, 21], (21,25], (25,35], (35,45], (45,55], (55,70], (70,∞))
BonusMalus	Numeric	Bonus/malus: <100 means bonus and >100 means malus in France (50-150)
VehBrand	Categorical	The car brand (B1-B14)
VehGas	Categorical	The car gas (diesel or regular)
LogDensity	Numeric	The log-density of inhabitants of the city where the policyholder lives (number of inhabitants per km^2^)
Region	Categorical	The policy region in France based on the 1970-2015 classification (22 classes)

**Table 5:** The distribution of the claim frequency and average claim severity.

Claim frequency	0	1	2	3	4	5	6	8	9	11	16
Number of policies	643953	23570	1299	62	5	2	1	1	1	2	1
Average claim severity	0	2177.12	2932.36	4115.35	2203.49	3559.01	1608.93	3103.22	2039.41	1966.92	2220.59

**Fig 5:**
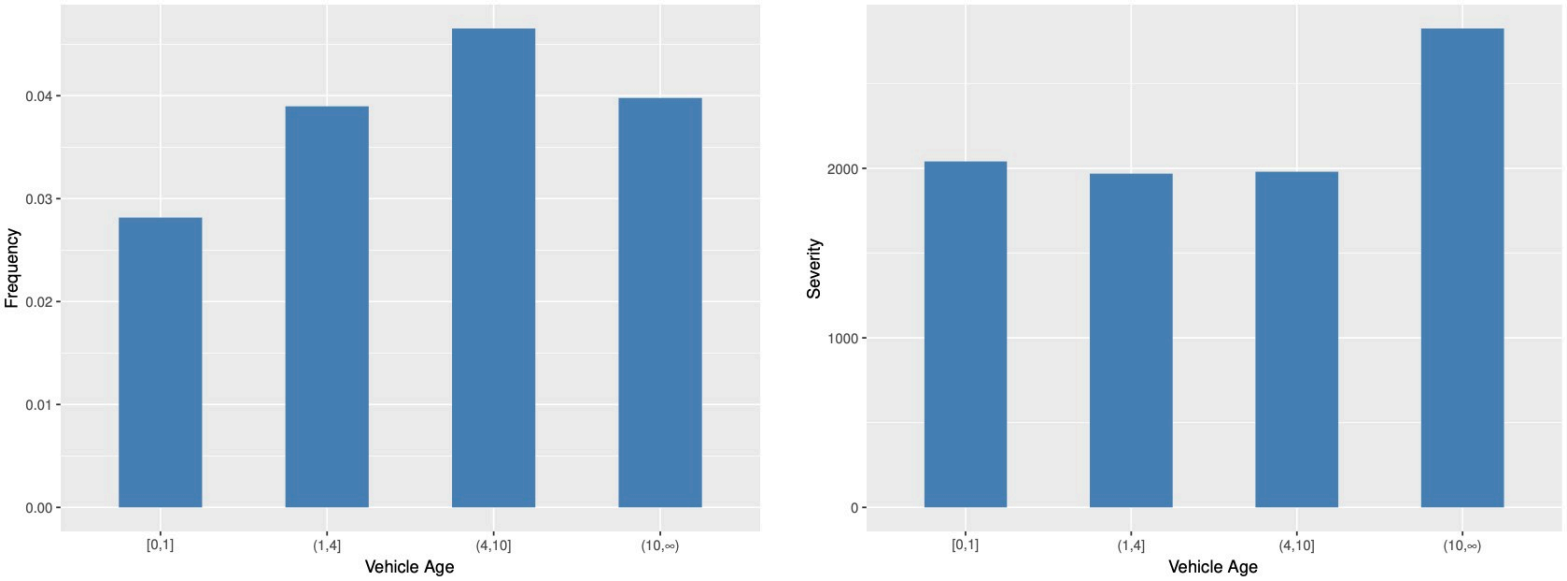
Histogram of the average claim frequency and severity per vehicle age group.

**Fig 6:**
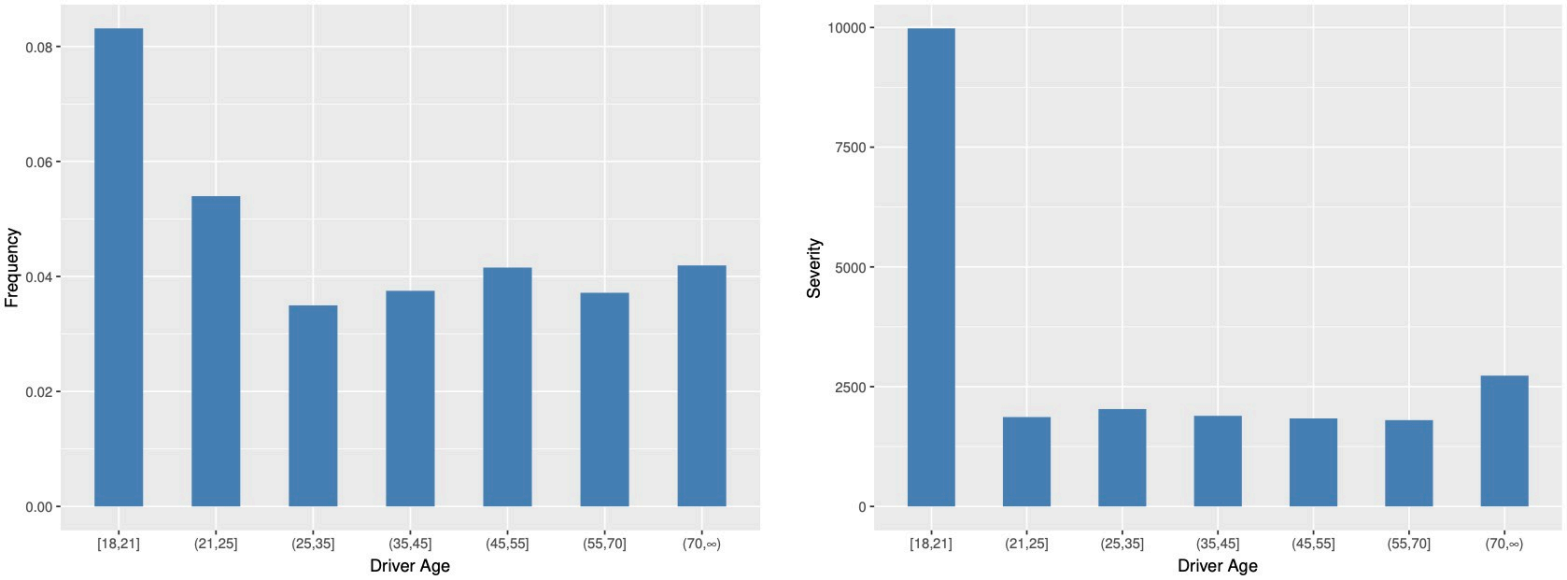
Histogram of the average claim frequency and severity per driver age group.

**Fig 7:**
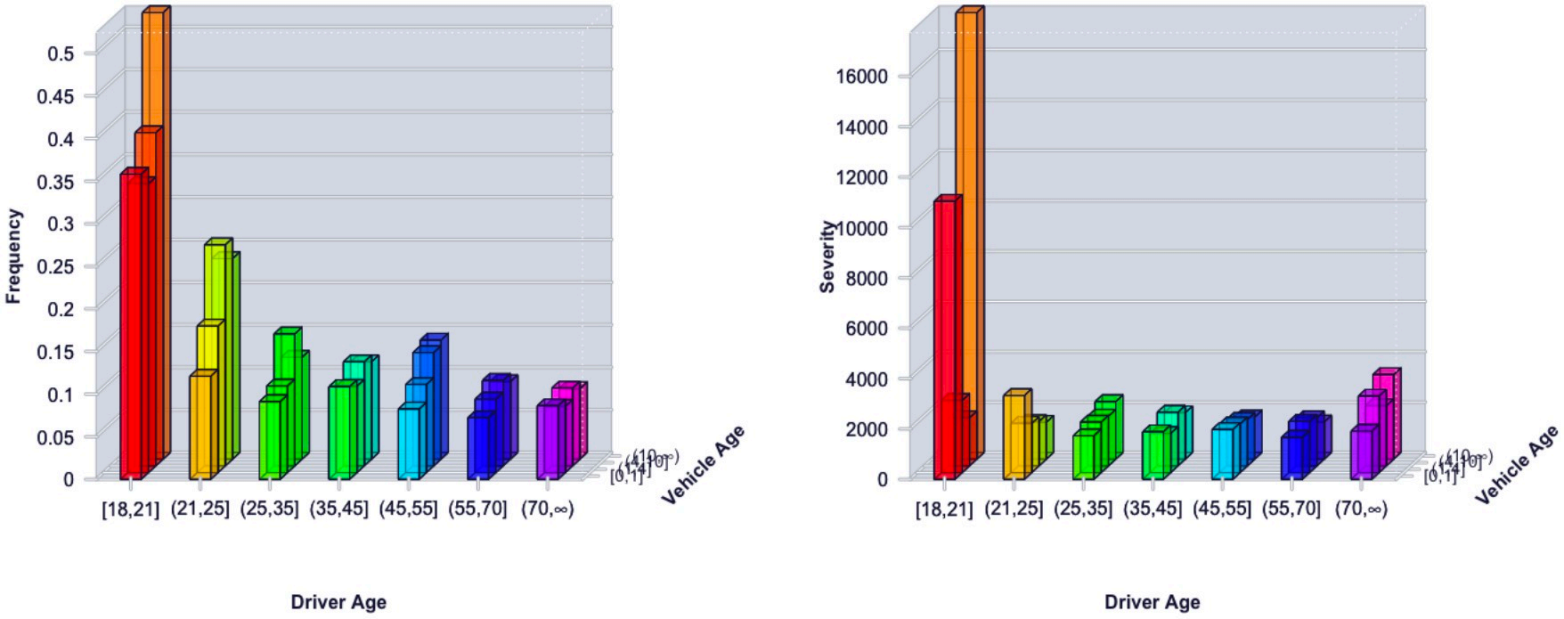
Histogram of the average claim frequency and severity per driver age and vehicle age group.

In Figs 5 and 6, we can find that the usage of old cars tend to incur more accidents and higher claim payments. Young drivers have less driving experience than middle-age and old drivers and cause more car crashes and more serious accident loss. In Fig 7, we can find that there are interactions among predictor variables. For young drivers, the vehicle age has a significant effect on the claim frequency. When the driver age increases, the effect gradually decreases. For young and old drivers, there are significant difference in the claim severity between different vehicle age groups. However, for middle-age drivers, the difference is small.

### Model comparison

We use 445931 observations as training data and the remaining 222966 as testing data. Then, we estimate the GLM, D-GLM, GAM, D-GAM, FSBoost and D-FSBoost models. In dependent models, we take the frequency/exposure instead of the frequency as the predictor variable. The FSBoost and D-FSBoost models can finish automatic feature selection. In the GLM, D-GLM, GAM and D-GAM models, we remove the insignificant variables. Table 6 shows out-of-sample loss for the models. The results indicate that dependent models are more competitive than independent models. The D-FSBoost model is most favorable.

**Table 6:** Out-of-sample loss.

	Frequency Loss	Severity Loss
GLM	-	82595.21
D-GLM	37664.53	82449.21
GAM	-	82108.26
D-GAM	34697.80	82008.89
FSBoost	-	78371.91
D-FSBoost	34155.54	78355.77

Then, we calculate pure premium prediction from the models on the testing data. We compare the models by using a Gini index to measure the discrepancy between the premium and loss distributions (Frees, Meyers, and Cummings [36, 37]). Let *B*(***x***) be the base premium and *T*(***x***) be the alternative premium. Denote by *Π*(***x***_*i*_) and *y*_*i*_ the pure premium and loss for the *i*^th^ observation, respectively. Frees, Meyers, and Cummings [36] define a relativity

$$\begin{array}{c} R\left(x\right)=\frac{T(x)}{B(x)} \end{array}$$ (41)

and order observations by relativities {*R*(***x***_1_), …, *R*(***x***_*θ*_)}. They define the ordered premium distribution as

$$\begin{array}{c} F_{\Pi }\left(s\right)=\frac{\sum_{i=1}^{\theta }\Pi \left(x_{i}\right)1_{R(x_{i})\leq s}}{\sum_{i=1}^{\theta }\Pi \left(x_{i}\right)} \end{array}$$ (42)

and the ordered loss distribution as

$$\begin{array}{c} F_{L}\left(s\right)=\frac{\sum_{i=1}^{\theta }y_{i}1_{R(x_{i})\leq s}}{\sum_{i=1}^{\theta }y_{i}}. \end{array}$$ (43)

The graph of (*F*_*Π*_(*s*), *F*_*L*_(*s*)) is an ordered Lorenz curve. When the percentage of losses equals the percentage of premiums, the curve results in a 45-degree line known as “the line of equality”. The Gini index is defined as twice the area between the Lorenz curve and the line of equality. Then, the empirical Gini index can be computed by

$$\begin{array}{c} \text{Gini}=1-\sum_{i=0}^{\theta -1}\left(F_{\Pi }\left(R\left(x_{i+1}\right)\right)-F_{\Pi }\left(R\left(x_{i}\right)\right)\right)\left(F_{L}\left(R\left(x_{i+1}\right)\right)+F_{L}\left(R\left(x_{i}\right)\right)\right), \end{array}$$ (44)

where *F*_*Π*_(*R*(***x***_0_)) = *F*_*L*_(*R*(***x***_0_)) = 0. A larger Gini index represents more profits for an insurer. Table 7 reports Gini indices calculated by using the prediction from each model as the base premium and using predictions from the remaining models as alternative premiums. Following Frees, Meyers, and Cummings [37] and Yang, Qian, and Zou [33], we use a “minimax” strategy to find the best model. For each base premium, we calculate the maximum Gini index over all alternative premiums. Then, we choose the base premium model that is least vulnerable to alternative premium models, i.e., we select the base premium model that has the smallest maximum Gini index. We find that the maximum Gini index is 0.9432 when using GLM as the base premium model, -0.1300 when using D-GLM, 0.0198 when using GAM, 0.0737 when using D-GAM, 0.0233 when using FSBoost, -0.2855 when using D-FSBoost. Thus, the D-FSBoost model represents the best choice.

**Table 7:** Gini indices.

**Base premium**	**Alternative premium**					
	**GLM**	**D-GLM**	**GAM**	**D-GAM**	**FSBoost**	**D-FSBoost**
GLM	-	0.0861	0.9432	-0.2309	-0.9472	-0.2004
D-GLM	-0.9999	-	-0.9998	-0.2126	-0.9995	-0.1300
GAM	-0.9939	-0.0996	-	0.0198	-0.9912	0.0101
D-GAM	-0.9999	-0.2779	-0.9998	-	-0.9995	0.0737
FSBoost	-0.9999	-0.1052	-0.9989	0.0233	-	0.0202
D-FSBoost	-0.9999	-0.3552	-0.9998	-0.2855	-0.9997	-

### Model interpretation

In this subsection, we use variable importance measures and partial dependence plots to interpret the D-FSBoost model. Variable importance measures show the importance of each predictor in predicting the frequency (severity). Partial dependence plots visualize the effect of the predictor on the frequency (severity).

#### Variable importance

For a single *K*−terminal node tree *T*_*i*_, Breiman, Friedman, Olshen, and Stone [38] introduce the following importance measure for the predictor *x*_*j*_,

$$\begin{array}{c} I_{x_{j}}\left(T_{i}\right)=\sum_{k=1}^{K-1}\rho_{k}1_{\upsilon_{k}}\left(x_{j}\right), \end{array}$$ (45)

where the sum is taken over all *K*−1 internal nodes, *υ*_*k*_ is the splitting variable in the node *k*, $1_{\upsilon_{k}}\left(x_{j}\right)$ is an indicator function that equals one when the splitting variable *υ*_*k*_ is *x*_*j*_, and *ρ*_*k*_ denotes the decrease in squared error by splitting the region associated with the node *k* into two subregions. Friedman [27] generalizes the variable importance measure to the gradient boosting model by taking the average over all trees {*T*_1_, …, *T*_*M*_},

$$\begin{array}{c} {\hat{I}}_{x_{j}}=\frac{1}{M}\sum_{m=1}^{M}I_{x_{j}}\left(T_{m}\right). \end{array}$$ (46)

The variable importance measure is biased since an independent predictor *x*_*j*_ can be selected as a splitting variable and hence ${\hat{I}}_{x_{j}}$ can not be zero. See Sandri and Zuccolotto [39, 40] for a bias correction.

**Fig 8:**
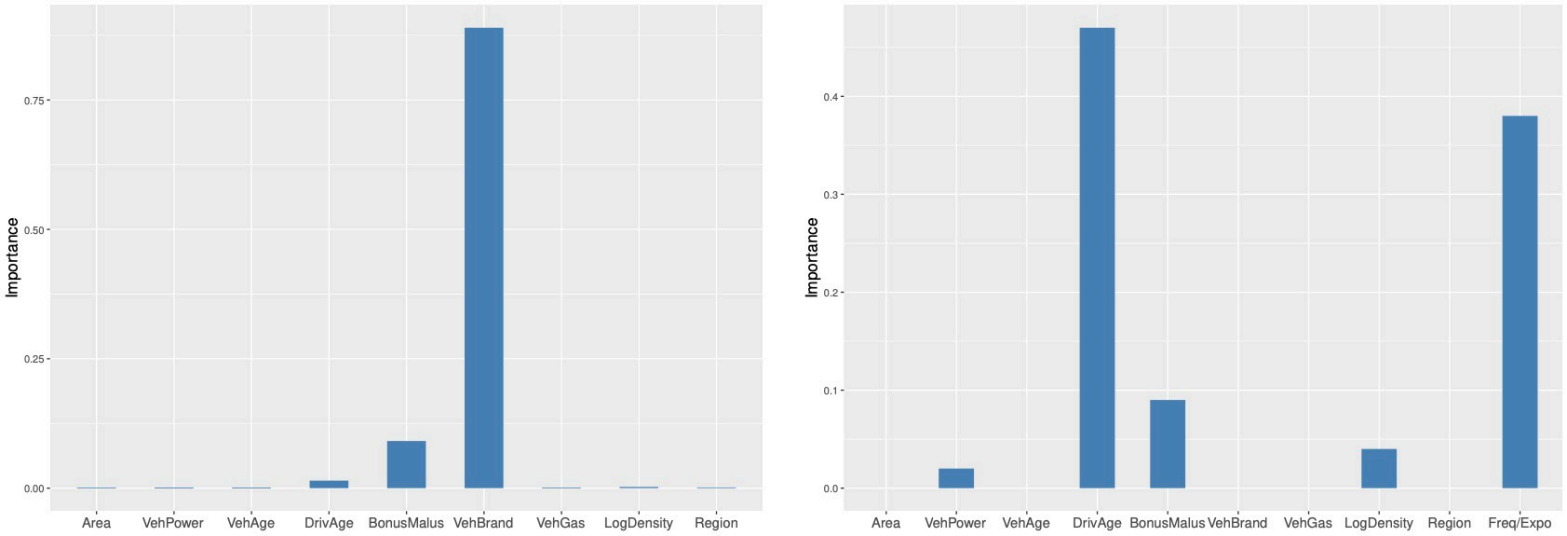
Variable importance measures.

In Fig 8, we show variable importance measures for the D-FSBoost model. We can find that VehBrand and BonusMalus are two most important variables in predicting the frequency. The VehBrand dominates the prediction. In predicting the severity, the variables DrivAge, Frequency/Exposure, BonusMalus and LogDensity are most influential. The DrivAge and Frequency/Exposure exert the leading effects. This result also provides further evidence on the dependence between the frequency and severity.

#### Partial dependence plots

Let ***z***_*k*_ be the subset of variables ***x*** and ***z***_−*k*_ be the complement subset of ***z***_*k*_ such that

$$\begin{array}{c} z_{k}\cup z_{-k}=x. \end{array}$$ (47)

The partial dependence of *F*_*N*_(***x***; ***α***) on ***z***_*k*_ can be calculated by

$$\begin{array}{c} \hat{F}\left(z_{k}\right)=\frac{1}{\theta }\sum_{i=1}^{\theta }F_{N}\left(z_{k},z_{i,-k};\alpha \right), \end{array}$$ (48)

where ***z***_*i*,−*k*_ is the *i*^th^ observation of ***z***_−*k*_. Then, the partial dependence plot of the frequency part is obtained by plotting the function $\hat{F}\left(z_{k}\right)$ against ***z***_*k*_. The partial dependence plot of the severity part can be obtained in the same manner.

**Fig 9:**
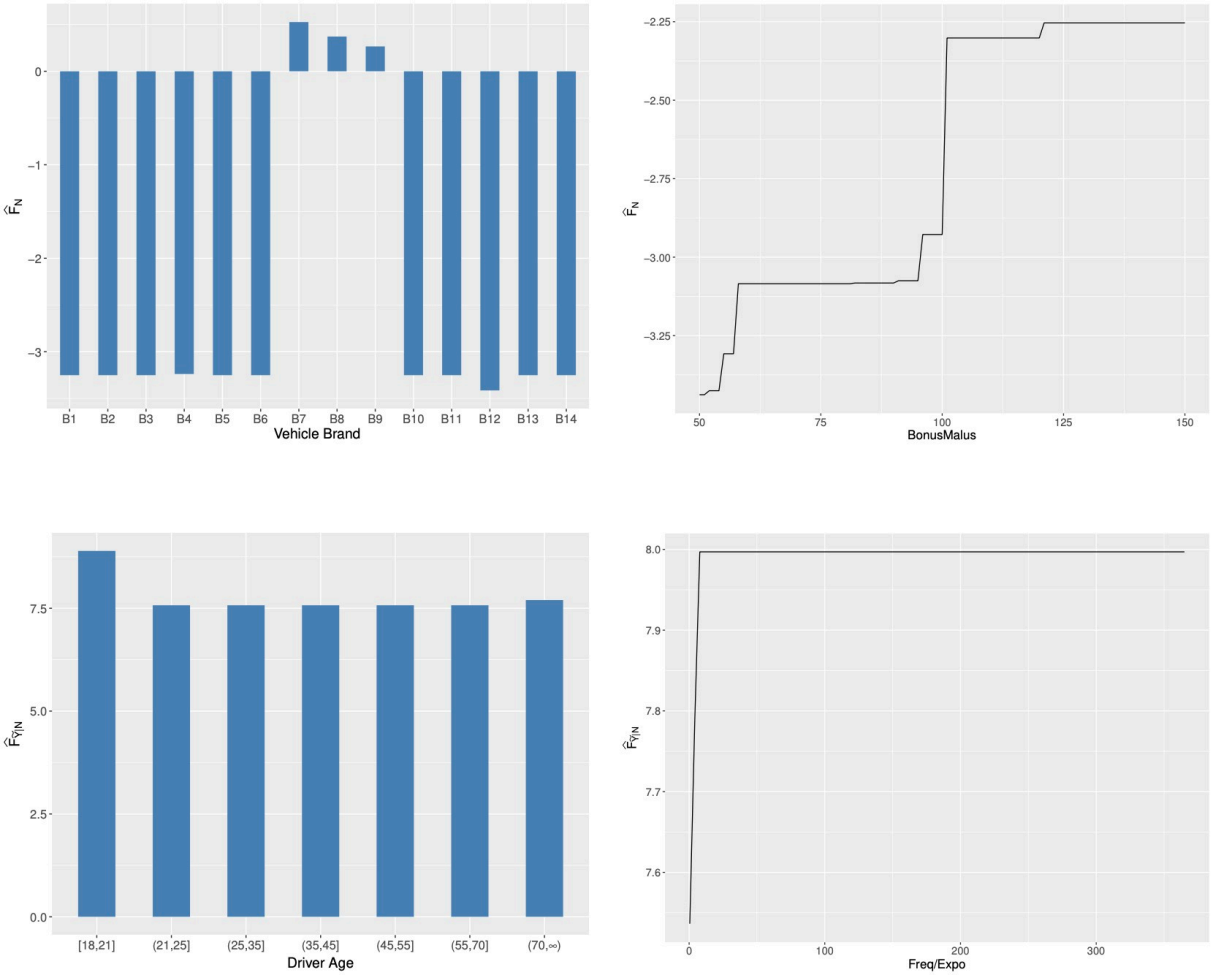
Partial dependence plots.

In Fig 9, we show the partial dependence plots for the D-FSBoost model, indicating the effects of two most important variables on the claim frequency and severity. From the top two panels, we can find that the car with brands B7-B9 causes much more accidents. The frequency is positively associated with the bonus-malus level. In France, the bonus-malus level less than 100 and larger than 100 means bonus and malus, respectively. The change from bonus to malus represents that at least an accident occurs. This explains the sudden change in the frequency at the bonus-malus level 100. The bonus-malus level 60 is the least bonus level to encourage policyholders to drive more carefully, which explains the sudden increase in occurrence of accidents when the bonus-malus level is near to 60. The bottom two panels show that young drivers induce more serious accidents. The severity increases dramatically when the claim frequency is small. This result is consistent with the observation in the distribution of the claim frequency and average claim severity.

## Conclusion

This paper develops a stochastic gradient boosting frequency-severity model by using the stochastic gradient boosting algorithm and profile likelihood approach. We demonstrate that the model can flexibly capture the nonlinear relation between the claim frequency (severity) and predictors and complex interactions among predictors, and can also fully capture the nonlinear dependence between the claim frequency and severity. The model is superior to other state-of-the-art models in the sense that it provides more accurate predictions in the claim frequency and severity distributions and pure premium.

In this paper, we illustrate the model with a Poisson distribution for the claim frequency and with a gamma distribution for the average claim severity. In fact, there are more flexible distribution choices. For example, we can use the negative binomial distribution for the claim frequency and the generalized gamma distribution for the average claim severity as in Shi, Feng, and Ivantsova [18]. The model can also be extended to capture different features of the claim data. For example, our model can combine with the hurdle and zero-inflated modeling framework to accommodate the overdispersion and zero inflation in the claim frequency. We can generalize our model to a random parameters version. We can also assume that the dispersion parameter depends on predictors and model the dispersion parameter with another ensemble of regression trees. These works are left for future research.

## Supporting information

S1 Dataset Motor. (CSV)
